# Prediction of Post-Operative Liver Dysfunction by Serum Markers of Liver Fibrosis in Hepatocellular Carcinoma

**DOI:** 10.1371/journal.pone.0140932

**Published:** 2015-10-26

**Authors:** Yinghao Shen, Guoming Shi, Cheng Huang, Xiaodong Zhu, Si Chen, Huichuan Sun, Jian Zhou, Jia Fan

**Affiliations:** Department of Liver Surgery, Zhongshan Hospital, Liver Cancer Institute, Fudan University, 180 Fenglin Road, Shanghai, China; The University of Hong Kong, HONG KONG

## Abstract

**Aim:**

To investigate the role of biomarkers in predicting postoperative liver dysfunction in patients with hepatocellular carcinoma (HCC).

**Methods:**

A total of 200 patients operated from July 2009 to June 2010 at Zhongshan Hospital, Fudan University for pathologically confirmed HCC were retrospectively analyzed for clinical data, HBD DNA level and serum biochemical markers for liver fibrosis. The patients were followed up to observersation end point. Correlation of the monitored parameters with postoperative liver dysfunction and patient survival was statistically analyzed.

**Results:**

Preoperative hepatitis B virus (HBV) DNA level, serum prealbumin (PA) hyaluronic acid (HA), and laminin (LN) levels correlated with postoperative liver dysfunction. A predictive model was generated using these 4 parameters and validated in 89 HCC patients with sensitivity and specificity of 0.625 and 0.912, respectively. However, no correlation was identified between postoperative liver function and overall survival.

**Conclusion:**

Liver fibrosis markers could be preoperatively used in predicting postoperative liver dysfunction in HCC patients.

## Introduction

Worldwide, the patients suffering from primary liver cancer are commonly complicated with cirrhosis, with a prevalence the latter of 72.1% to 82.3%[1]. Compared to non-cirrhotic ones, the cirrhotic patients have relatively poor liver regeneration ability and impaired functional reservation. A number of extensive studies have shown that liver dysfunction or liver failure contributes to the majority of postoperative mortality of HCC. As such, accurate preoperative assessment of liver reserve function is important for not only safety of liver surgery but also post-operative survival.

A variety of methods are currently used to estimate liver function reserve, including liver enzyme tests, indole indocyanine green clearance test (ICG15), liver elasticity index detector, nuclear imaging and Child-pugh grade. However, no consensus has been reached yet regarding superiority of any one among these methods over the others[2].

Hepatitis B virus infection is prevalent in Asian countries. In China, the majority of patients with primary liver cancer have HBV infection leading to cirrhosis[3]. Diagnosis criteria of liver fibrosis, the reversible precursor of cirrhosis, includes histopathology, imaging and serum markers, with pathology serving as the gold standard[4]. Besides, emerging as a new technology for accessing liver fibrosis, liver stiffness measurement (LSM) determines transient elastography (TE)[5]. Accumulating clinical data have shown that LSM is slightly superior to Fibrotest and AST-to-Platelet Ratio Index (APRI) in terms of diagnosis efficacy[6]. However, LSM requires highly trained operators, and is adversely affected by abdominal obesity statue/body mass index (BMI)[7], liver inflammation activity, and abnormalities of bilirubin or transaminase as well[8, 9], and thus does not gain clinical popularity yet.

Serum markers for liver fibrosis including hyaluronic acid (HA), laminin (LN), IV collagen (IV-C) and Ⅲ procollagen N-terminal peptide (P III-NP) have been recently considered as important evaluation of disease progress, inflammatory activity, fibrosis and therapeutic effect in chronic liver diseases[10–12]. Pathophysiologically, along with the pathological process of liver fibrosis, balance between synthesis and degradation of extracellular matrix in liver tissue (ECM) is disturbed, leading to excessive proliferation and abnormal deposition of ECM, which is reflected by release into serum of ECM components/degraded products, collagenases and certain cytokines. Among the four above-mentioned serum markers of ECM components/degraded products P III-NP and IV-C represent extracellular matrix collagen, reflecting basement membrane collagen metabolism, while HA and LN mirror metabolism of basement membrane glycoproteins. Authors believe that these four markers can more accurately reflect the metabolic changes in the liver, including fiber formation, degradation, deposition, of extracellular matrix components.

Herein we reported retrospective evaluation of role of the four indicators in predicting postoperative liver dysfunction and death of liver failure in patients with primary liver cancer. We identified risk factors affecting postoperative liver function recovery in these patients, and also generated an easy-to-use mathematical model for estimating liver functional reserve preoperatively on this basis.

## Materials and Methods

### Study subjects

Clinical data of a total of 200 patients operated from July 2009 to June 2010 at Zhongshan Hospital, Fudan University for pathologically confirmed hepatocellular carcinoma were collected, with those with obstructive jaundice, biliary disease, hepatitis C and alcoholic cirrhosis and incomplete data being excluded. These patients were followed up till end of 2012. Another 89 cases meeting the above criteria and admitted between June 2010 to November 2010 were selected for validation of the mathematical model. This study was approved by The Institutional Review Board of Ethics Committee of Zhongshan Hospital, Fudan University. All participants received written and oral information prior to giving written consent, and the study was performed in accordance with the Helsinki II declaration.

### Diagnostic criteria

The 2011 International Liver Surgery Group (ISGLS) liver function decompensation criteria was adopted in the present study[13].

### Biochemical tests

HA, LN, P III-NP and IV-C were measured by magnetic microbead chemoluminence method following the manufacturer’s manual (Antu Bioengineering Co Ltd, Zhengzhou, China). All other tests were routine analysis done at our clinical laboratory.

### Clinical information

Clinical data including age, sex, accompanying diabetes, HBV-DNA copy number, alpha-fetoprotein (AFP), Child-Pugh score, total bilirubin (TB), bilirubin (CB), serum albumin (Alb), alanine aminotransferase (ALT), alkaline phosphatase (ALP), γ- glutamyl GGT (γ-GT), prealbumin (PA), prothrombin time (PT), HA, LN, P III-NP, IV-C, tumor size, procedure type and total hepatic hilar occlusion time were collected and recorded.

### Statistical Methods

Data with gaussian distribution were presented as mean ± standard deviation, and analyzed with independent sample t-test. Skewed variables data were presented as median (range) and analyzed with non-parametric Wilcoxon test. Attributes data were analyzed with chi-square test. Multivariate analysis was done using logistic regression with the indicators of p <0.1 in univariate analysis included, and backward method based on the likelihood ratio test of conditional parameters was used in this analysis, in which the variable was removed out of the equation when p> 0.05. The variables selected by logistic regression analysis were employed to establish an equation calculating the postoperative liver failure risk following published approach [14–16]. In this model, each dichotomous variable was multiplied by its regression coefficient. The resulting products were then added up to generate the risk score of each patient. Calculating the area under curve of the ROC was performed using non-parametric method. Survival data were analyzed by Log-rank test. Difference with P<0.05 was considered as statistically significant.

## Results

### Preoperative levels of liver fibrosis markers in the patients with HCC

**Fig 1:**
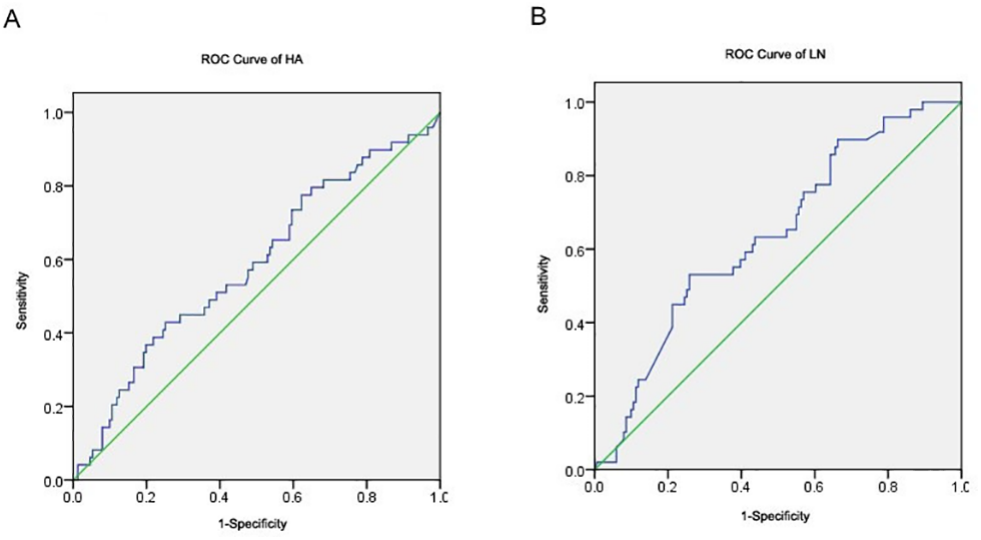
ROC curves of pre-operative HA and LN in evaluating postoperative liver dysfunction. A, ROC curve of HA; B, ROC curve of LN.

Preoperative HA, LN levels were analyzed using ROC curve and further stratified using Youden index selection method in which cut-off points were picked based on the largest index numbers. Specifically, HA had a cut-off value of 139 ng/ml with sensitivity of 0.557 and 1-specificity of 0.366. And LN had a cut-off value of 485 ng/ml, with sensitivity of 0.531 and specificity of 0.742 (Fig 1). Other conventional biochemical measurements were interpretated based on their reference values.

Statistical analysis also showed that significant differenence existed between post-operatively liver function compensated and decompensated patients in preoperative AFP, HBV-DNA, TB, γ-GT, PT, HA, PA and LN levels, while no difference existed between these 2 groups of patient either in age, gender, diabetes, Alb, ALT, P III-NP, IV-C, tumor size, extent of resection or hilar occlusion time (Table 1).

**Table 1:** Preoperative measurements in the liver function compensated and decompensated patients.

Index	Compensated(n = 151)	Decompensated(n = 49)	T/ χ^2^ /Z	P
Age(year, χ±s)	53.62±11.71	53.20±9.54	0.223	0.824
Gender				
M	128	44	0.777	0.378
F	23	5		
Diabetes				
(-)	141	46		
(+)	10	3		1.000
Child-pugh				
A	79	168	2.498	0.114
B	3	17		
Score	5.11±0.423	5.31±0.728	-2.318	0.022
AFP(ug/L)				
≤20	63	12	4.687	0.041
>20	88	37		
HBVDNA				
≤103/mL	88	15	11.337	0.001
>103/mL	63	34		
TB(umol/L)				
< = 20.4	56	7	8.913	0.003
>20.4	95	42		
Alb (g/L)				
≤40	84	34	2.895	0.097
>40	67	15		
ALT(U/L)	30.30(300)	38.80(95)	-1.108	0.308
γ-GT(U/L)				
≤45	52	9	4.507	0.034
>45	99	40		
PA (g/L)	0.21±0.048	0.18±0.051	4.392	<0.001
PT (s)	11.80±0.88	12.16±1.03	-2.42	0.016
HA (ng/ml)				
≤139	113	28	5.568	0.018
>139	38	21		
LN (ng/ml)				
≤485	112	23	12.507	<0.001
>485	39	26		
P III-NP(ng/ml)				
≤15	120	35	1.372	0.241
>15	31	14		
IV-C (ng/ml)				
≤95	95	24		
>95	56	25	2.981	0.084
Tumor size				
≤3cm	54	14	0.852	0.39
>3cm	97	35		
Resected liver segments				
<3	110	31	1.633	0.201
≥3	41	18		
Hilar occlusion(min)	10(108)	8(45)	-0.518	0.605

### Determinants of postoperative liver dysfunction

As shown in Table 2, Patients with higher HBV-DNA copy number had 2.96 times of risk developing postoperative liver dysfunction comparing to the patients with lower HBV viral load. Similar results were observed with HA and LN measurements. As for TB, a level greater than 20.4 umol/L imposed on the patient 4.294 times of such risk comparing those with TB<20.4 umol/L. In terms of PA, a decrease of 0.1g/L brought up odds ratio of liver function insufficiency to 3.91(1/0.256).

**Table 2:** Logistic analysis of postoperative liver dysfunction.

	B	S.E,	Wals	p	OR	OR 95% C.I.
HBVDNA(control: ≤103mL)	1.085	.398	7.427	.006	2.960	1.356–6.460
TB(control: ≤20.4)	1.457	.496	8.619	.003	4.294	1.623–11.361
PA	-1.364	.427	10.180	.001	.256	.111-.591
HA(control: ≤139)	.883	.411	4.604	.032	2.417	1.079–5.412
LN(control: ≤485)	1.018	.387	6.915	.009	2.767	1.296–5.908

### Mathematical model for estimating postoperative liver dysfunction

Based on logistic regression analysis results, an equation was generated for quantitatively evaluate risk of postoperative liver dysfunction:

Y (postoperative liver failure risk) = 1.2414 × (1 for HBV DNA> 10^3^/mL, otherwise 0) + 1.667 × (1 for TB> 20.4 umol / L, otherwise 0) -1.5606 × PA + 1.0103 × (1 when HA> 139 ng / ml otherwise 0) + 1.1648 × (1 when LN> 485 ng / ml, otherwise 0).

**Fig 2:**
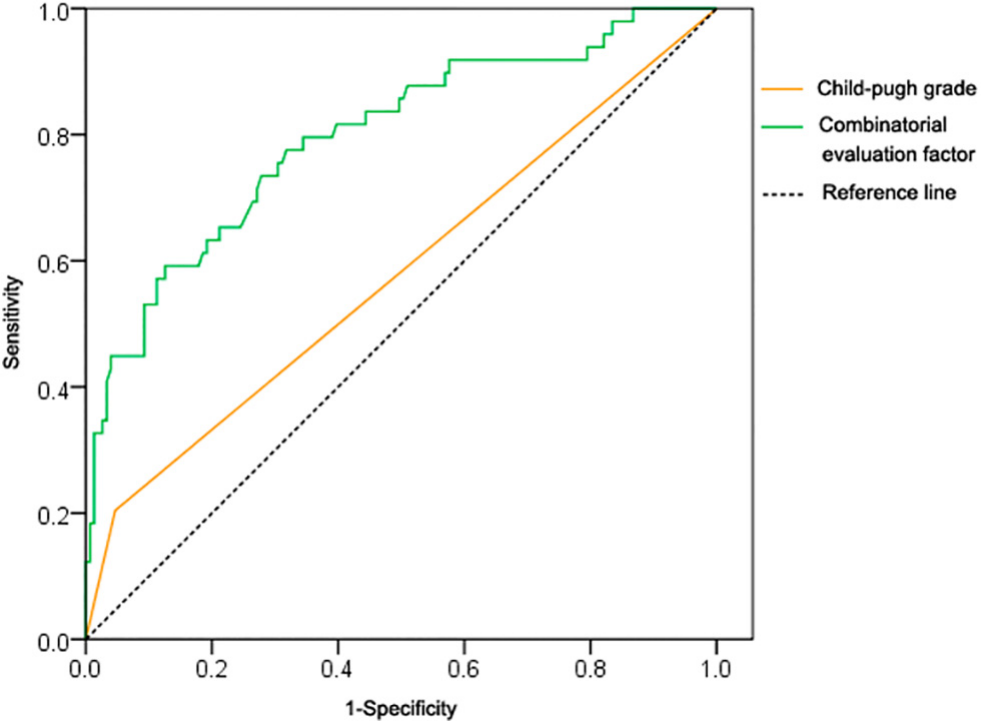
ROC curve of the combinatorial evaluation factor and Child-Pugh in estimating postoperative liver dysfunction.

The area under curve of Y was 0.797 (95% CI = 0.721–0.873), while Child-pugh grade had an area under curve of 0.579 (95% CI = 0.481–0.676), statistically smaller than that of Y (p <0.05), indicating that the combinatorial evaluation factor outperformed the Child-pugh classification. The cut-off point of Y values was 0.2704, with sensitivity and specificity of 0.592 and 0.874, respectively (Fig 2).

### Validation of the mathematical equation

The validation data demonstrated that, 25 of the 89 cases had Y values above the threshold, and 80% (20 of 25) developed postoperative liver decompensation. Among the 64 with Y values below the threshold, 12 (18.75%) had liver decompensation with no death. The sensitivity and specificity of the model was 0.625 and 0.912, respectively (Table 3).

**Table 3:** Validation of the mathematical equation.

	Postoperative liver dysfunction		
	Presence	Absence	Total (n)
Dysfunction not predicted	52	12	64
Dysfunction predicted	5	20	25
Total (n)	57	22	89

### Survival analysis

**Fig 3:**
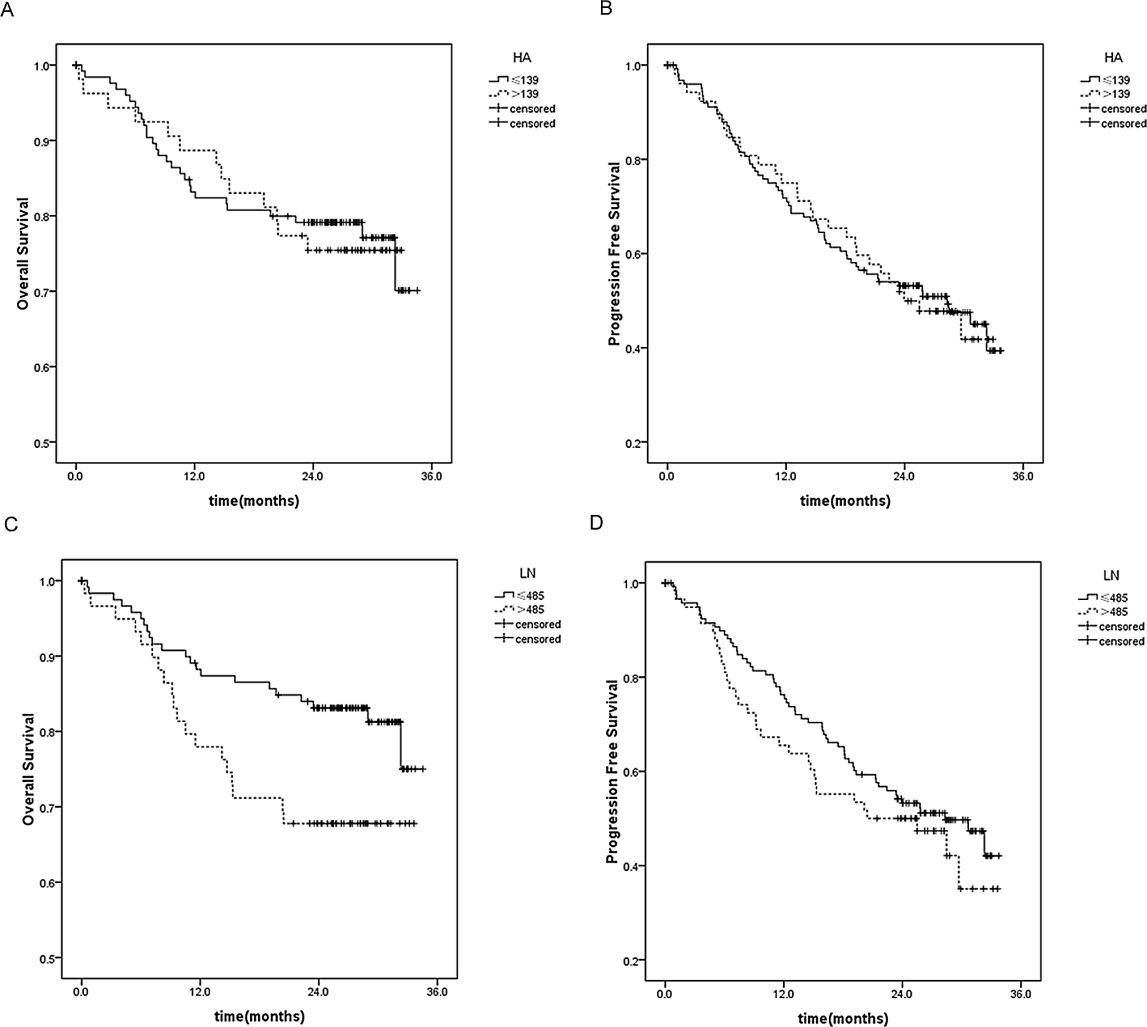
Survival of the patients stratified on basis of HA and LN. A, Overall survival after stratification with HA level (χ^2^ = 0.048, p = 0.827); B. Tumor-free survival after stratification with HA level (χ^2^ = 0.006, p = 0.937); C, Overall survival after stratification with LN level (χ^2^ = 4.698, p = 0.03); D, Tumor-free survival after stratification with LN (χ^2^ = 1.077, p = 0.299).

The patients were stratified with levels of HA and LN for survival analysis (Fig 3). The 24-month survival rates were 0.83 and 0.68, respectively. However, no correlation was identified between postoperative liver function and overall or tumor-free survival (P all >0.05).

## Discussion

Hepatectomy is treatment of choice for HCC and estimation of hepatic functional reserve of the future remnant liver is critical for liver surgery. Along with the continuous improvement in preoperative evaluation, surgical techniques and perioperative management, refinement of liver surgery brings postoperative mortality to less than 5%. However, post hepatectomy liver failure (PHLF) associated death is still the main source of mortality in the short period after surgery[17, 18]. Thus, accurate assessment of liver functional reserve and prediction of postoperative liver dysfunction is essential for hepatectomy planning. Conventionally, pre-operative assessment of liver function usually includes biochemical tests, liver function scoring systems, quantitative hepatic functional analyses, imaging evaluation, regional radiographic assessment of liver function and liver volume measurements. However, none of these methods are accurate and reliable. In fact, people tend to apply multiple methods for comprehensive assessment with hope to improve accuracy of liver reserve function evaluation. For example, Clavien et al integrated histopathology, Child-pugh score, ICGR15 and status of portal hypertension in their prediction of resection volume of liver, which yielded successful clinical outcomes. In addition, recent study reveals that besides extent of hepatectomy, preoperative HBV DNA level, high Ishak fibrosis score and HBV reactivation are important prognostic factors for postoperative liver dysfunction as well. In the present study, liver dysfunction was present in 49 cases, including ISGLS A grade in 25 cases, grade B in 22 cases, grade C in two cases. The two 2 patients in grade C died of failure, while the remaining 47 patients recovered after appropriate management. Besides, our data showed that the liver fibrosis markers, i.e., HA, and LN, were significantly shifted in post-operatively decompensated patients. Moreover, our data suggested that pre-operative LN level predicted post-operative survival in this population. Our comprehensive prediction model taking HBV DNA copy number, total bilirubin, PA and HA into consideration had sensitivity and specificity of 0.592 and 0.874, respectively. Taken together, our results demonstrated that combination of liver fibrosis markers with HBV DNA and total bilirubin could be an easy and practical approach assessing liver function for hepatectomy.

Serum total bilirubin is widely used to assess liver injury. During cirrhosis process, the regenerated hepatocytes are not connected to primary or proliferated and the conjugated bilirubin enters directly into bloodstream, causing bilirubinemia. As a matter of fact, serum total bilirubin level has been considered as a predictor of postoperative death caused by PHLF[18, 19]. In Castera’s retrospective study, preoperative serum bilirubin ≥20.4umol/L and serum prealbumin <0.14 g/L are independent risk factor for postoperative liver dysfunction for hepatectomy in HCC patients[20]. Consistent with this finding, we found that preoperative TB>20.4 umol / L carried much higher risk for postoperative liver dysfunction than that of TB<20.4 umol /L. Similarily, Yachida S et al reported that total serum bilirubin ≥17umol/L and blood hyaluronic acid ≥120ng/mL have the same significance.

Cirrhosis is another important predictor for poor prognosis after hepatectomy in HCC[21]. Accumulating clinical data indicate that serum liver fibrosis markers (HA, LN, IV-C and P III-NP) are efficient indicators of severity of liver fibrosis[22–26]. Although studies have shown that preoperative serum HA correlates well with postoperative liver dysfunction, but the HA thresholds were set at different levels in different studies[27–29]: It was set at 200 ng/mL in Ogata’s series[25] but 100 ng/mL in Mima’s study[20], and we used HA>139 ng/ml as cutoff. Apparently, the variation in sensitivity of HA measurement necessitates further clinical studies for a more accurate reference level with satisfactory sensitivity and specificity.

LNs are 400 kD extracellular matrix glycoproteins binding to type IV collagen to form network of basal membrane for regulation of intercellular cell adhesion, migration, and cell growth/differentiation, and influencing cirrhosis and tumor spread. LNs are mainly expressed in vessel wall, bile duct and lymphatic wall, but little in normal hepatocytes[30]. They are over-expressed in liver fibrosis and deposited in sinusoidal endothelial cell gap, which reduces permeability of endothelial cells, and consequently leading to increased portal pressure. Elevated LN level indicates fibrosis of the sinusoidal capillaries and portal triad[31–33]. Using 485 ng/ml as a cut-off, we found that high LN level was significantly associated with not only post-hepatectomy liver dysfunction but also shortened 24-month overall survival. As such, we believe that preoperative high LN level is a good predictive marker for postoperative liver dysfunction and general prognosis in HCC.

By logistic analysis, we were able to generate a mathematical model for predicting postoperative hepatic dysfunction. The model contained components of HBV-DNA, serum total bilirubin, HA and LN. It was verified with clinical data from 89 patients with sensitivity and specificity of 0.625 and 0.912, respectively. Obviously, there is room for improvement of the sensitivity of the model while the specificity was favorable. Nevertheless, comparing to that of Child-pugh grades alone, our model had improved diagnostic performance in general.

The present study has a couple of limitations. First, this was a retrospective study. Second, other clinical measurements such as indole indocyanine green clearance test (ICG15) and liver elasticity index testing were not in the scope of the study. Further prospective studies are needed to confirm our findings and improve performance of the prediction model for assessing postoperative liver dysfunction in HCC.
