# Correction: Prediction of Post-Operative Liver Dysfunction by Serum Markers of Liver Fibrosis in Hepatocellular Carcinoma

**DOI:** 10.1371/journal.pone.0145141

**Published:** 2015-12-11

**Authors:** The PLOS ONE Staff

There is an error in the third sentence of the Results section of the Abstract. The correct Results section of the Abstract should read: Preoperative hepatitis B virus (HBV) DNA level, serum prealbumin (PA) hyaluronic acid (HA), and laminin (LN) levels correlated with postoperative liver dysfunction, and preoperative LN level also was associated with the patients’ survival. A predictive model was generated using these 4 parameters and validated in 89 HCC patients with sensitivity and specificity of 0.625 and 0.912, respectively.

There is also an error in the Survival Analysis section of the Results. The correct Survival Analysis section should read: The patients were stratified with levels of HA and LN for survival analysis (Fig 3). Log-rank test results showed that difference in overall survival was significant only when patients were stratified with LN (p <0.05). The 24-month survival rates were 0.83 and 0.68, respectively.

The publisher apologizes for these errors.
